# Are In Vitro Cytotoxicity Assessments of Environmental Samples Useful for Characterizing the Risk of Exposure to Multiple Contaminants at the Workplace? A Systematic Review

**DOI:** 10.3390/toxics10020072

**Published:** 2022-02-05

**Authors:** Carla Viegas, Pedro Pena, Bianca Gomes, Marta Dias, Liliana Aranha Caetano, Susana Viegas

**Affiliations:** 1 H&TRC—Health & Technology Research Center, ESTeSL—Escola Superior de Tecnologia da Saúde, Instituto Politécnico de Lisboa, 1990-096 Lisbon, Portugal; pedro_migpena@hotmail.com (P.P.); bianca.gomes@estesl.ipl.pt (B.G.); marta.dias@estesl.ipl.pt (M.D.); liliana.caetano@estesl.ipl.pt (L.A.C.); susana.viegas@ensp.unl.pt (S.V.); 2 NOVA National School of Public Health, Public Health Research Centre, Universidade NOVA de Lisboa, 1099-085 Lisbon, Portugal; 3 Comprehensive Health Research Center (CHRC), NOVA Medical School, Universidade NOVA de Lisboa, 1169-056 Lisbon, Portugal; 4 Research Institute for Medicines (iMed.ULisboa), Faculty of Pharmacy, University of Lisbon, 1649-003 Lisbon, Portugal

**Keywords:** multiple contaminants, environmental samples, in vitro studies, cytotoxicity, occupational health, risk management

## Abstract

In some occupational environments risk characterization is challenging or impossible to achieve due to the presence of multiple pollutants and contaminants. Thus, in vitro testing using the most relevant cell lines will provide information concerning health effects due to the co-exposure to multiple stressors. The aim of this review article is to identify studies where the cytotoxicity assessment was performed in environmental samples, as well as to describe the main outputs and challenges regarding risk characterization and management. This study is based on a study of the available information/data on cytotoxicity assessment performed on environmental samples following the PRISMA methodology. Different cell lines were used depending on the environment assessed and exposure routes implicated. The A549 alveolar epithelial cell line was applied in four studies for occupational exposure in the waste sorting industry and for outdoor environments; lymphocytes were used in two studies for occupational and outdoor environments; swine kidney cells were used in three studies performed in the waste industry and hepatocellular/Hep G2 in one study in the waste industry. Cytotoxicity assessments in environmental samples should have a more prominent role due to their contribution for identifying and better understanding the associations between co-exposure to environmental contaminants and adverse human health effects as a prioritization for risk management.

## 1. Introduction

In specific occupational environments, due to the presence of multiple pollutants and contaminants, risk characterization is very demanding, if not impossible, to accomplish. As an example, dust exposure is a major source of harmful respiratory outcomes among agricultural workers [1,2]. Dairy farm dusts are complex mixtures of chemicals, microorganisms and their metabolites [3], and endotoxins their most well characterized contaminant [4]. However, the inflammatory potential of such dusts does not depend merely on endotoxins, since dust composition varies between workplaces, tasks and occupational environment [5,6].

Traditionally, the one pollutant-at-a-time approach has been applied both for hazard characterization and exposure assessment, neglecting the health effects caused by human exposure to mixtures [7]. Even with refined sampling and analyses protocols, the most frequent situation is one where researchers have insufficient knowledge about the pollutants present in the mixture [8]. However, exposure to complex mixtures in occupational settings is the most common exposure scenario [7,8,9].

In vitro studies can be used as a first line screening tool to characterize the co-exposure to multiple stressors and the biological effects of mixtures present in environmental samples [8,10], within the scope of European Union directives to reduce and refine animal toxicity testing. The characterization of in vitro toxicity is based on the assessment of biological responses, such as cellular cytotoxicity or DNA damage, by exposing relevant cells or cell lines to complex mixtures of pollutants and contaminants, as with samples from different indoor environments in exposure assessment studies [8]. An advantage of in vitro toxicology is the estimation of health effects based on the biological responses observed, even if the detailed characterization of the combined mixture of pollutants present in the environmental sample is not known [7]. A limitation is that, among studies devoted to health effects associated with simultaneous exposure to multiple pollutants and contaminants, very few report quantitative estimates of combined health effects [8]. These quantitative estimates would contribute to a better risk characterization and prioritization of action, as well as to a selection of the most appropriate risk management measures [11].

The aim of this systematic review was to identify the in vitro approaches (including sample type, cellular system) most commonly used for the cytotoxicity assessment of complex environmental samples from different indoor settings, and to describe the main outputs and challenges regarding risk characterization and management.

## 2. Materials and Methods

### 2.1. Registration

The protocol of this systematic review was submitted for registration in PROSPERO (ID Number: 290440). Moreover, the Preferred Reporting Items for Systematic Reviews (PRISMA) checklist was completed (Table S1—Supplementary Material).

### 2.2. Search Strategy and Inclusion and Exclusion Criteria

This study is based on a review following the PRISMA methodology of the available information/data on cytotoxicity assessments performed on environmental samples, published between the 1 January 2000 due the recently increase of in vitro resources and the 31 May 2021. The databases chosen were PubMed, World of Science and Scopus, and the keywords used were “cytotoxicity” AND “cytotoxicity effects” AND “human health” AND “occupational exposure” AND “occupational health” AND “public health” AND “exposure assessment” AND “environmental health” OR “environmental isolates”. Searches were carried out in English and articles that did not meet the inclusion criteria were excluded from further analysis (but some of them were used for introduction and discussion sections; Table 1).

### 2.3. Study Selection and Data Extraction

The selection of the articles was performed in two rounds by three investigators (PP, BG and MD). The first round consisted of a screening of all titles and abstracts. In the second round, the full texts of all potentially relevant studies were reviewed considering the inclusion and exclusion criteria. Potential divergences in the selection of the study were discussed and ultimately resolved by the remaining investigators (CV, LAC and SV). Data extraction was performed by one investigator (PP/BG/MD) and reviewed by the other two. The following information was manually extracted: (1) title, (2) country analyzed, (3) environment samples description/number of samples, (4) cell lines applied and (5) main findings.

### 2.4. Quality Assessment

The assessment of the risk of bias was performed by three investigators (CV, LAC and SV). Within each study, we evaluated the risk of bias across three parameters divided as key criteria (environment samples description/number of samples, cell line applied and main findings) and other criteria (incomplete data about cell lines and conflict of interest). The risk of bias for each parameter was evaluated as “low”, “medium”, “high”, or “not applicable”. The studies for which all the key criteria and most of the other criteria are characterized as “high” were excluded.

## 3. Results

**Figure 1:**
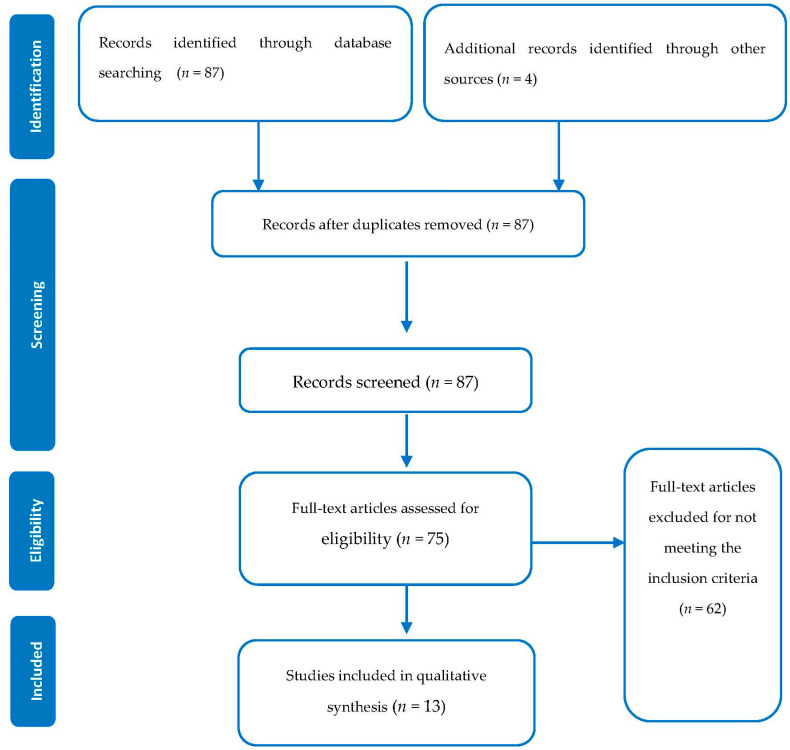
PRISMA methodology of selection of papers and other sources.

Figure 1 shows the flow diagram for the selection of the studies. The primary search on the databases returned 87 studies and four additional studies from other scientific sources, from which 91 abstracts were screened, and 87 full texts were assessed for eligibility. After considering the inclusion and exclusion criteria, a total of 62 papers were excluded mainly because they were either related to biological samples or performed cytotoxicity evaluation only on biological samples. A total of 13 articles about cytotoxicity assessment were finally selected.

### Characteristics of the Selected Studies

Table 2 describes the main characteristics the selected studies. Most of the studies (six out of 13) analysed environmental samples from occupational environments, such as waste sorting industries (4), dairy farms (1), slaughterhouses (1). Outdoor environments (six out of 13) and indoor environments (one) were also analysed. The most frequent sampling methodology used was passive sampling (eight out of 13). Active air sampling was performed (four out of 13) using different samplers depending on the pollutants that needed to be assessed. Air sampling through the impaction method where a specific flow rate is defined to collect air particles on a collection media by promoting particle separation through an air stream (two out of 13) and particulate sampling through filters (two out of 13) were the most frequently reported techniques. Additionally, two studies collected filtering respiratory protection devices and their interior layer and exhalation valve; one study collected filters from the heating, ventilation and air conditioning system from forkliftes operating in a waste sorting facility; one sampled mechanical protection gloves used in waste sorting and other two performed water sampling (Table 2).

Different cell lines were used, depending on the environment assessed and exposure routes implicated. The A549 alveolar epithelial cell line was used in four outdoor environment studies [12,13] and for occupational exposure in the waste sorting industry [11,14]. Lymphocytes were used in two studies of occupational and outdoor environments [15,16]. Swine kidney cells were used in three studies in the waste industry [11,14,17], and hepatocellular/Hep G2 cells in one study in the same environment [17]. Human bronchial epithelial (NHBE or BEAS-2B) cells were used in three studies for occupational [4], indoor [18] and outdoor environments [19], respectively. Other cells lines, such as THP-1 and HUVEC cells, were used in two studies in waste sorting [17] and kitchen environments [20].

After exposure of cells to samples’ extracts, most of the studies (nine out of 13) reported cytotoxic effects, with reduction of cell viability (two out of 13), inflammation (one), and oxidative stress (one) being some of the alterations identified. Proinflammatory responses were also recurrent (five out of 13), with a singular study revealing pro-inflammatory response in airway epithelial cells and others revealing the production of TNFα (1). Genotoxicity was also observed (three out of 13 studies), being associated with DNA damage in lymphocytes (two). As complementary data, one study reported the buccal micronucleus cytome (BMCyt) assay as a good, non-invasive biomarker of cyto-genotoxicity in target organs (Table 2).

## 4. Discussion

Occupational environments dedicated to waste sorting were the ones more reported in this review. In fact, due to the challenges in this occupation, and in the other settings mentioned in this review, to accurately assess all the pollutants present, the cytotoxicity assessments performed can have an added value regarding risk characterization, as well as prioritizing interventions to minimize exposure [11,14,17,21]. Furthermore, the overall effects on workers’ health is a complex endeavour, and will strongly depend on tasks performed and exposure levels of the pollutants present in the occupational environment, genetic factors and the workers’ individual innate immune defence [22,23,24].

Further, and focusing on the waste industry, the change from a linear economy (take, make, dispose) to a circular economy (renew, remake, share) is expected to support significantly the attainment of the Sustainable Development Goals (SDGs) from United Nations (https://sdgs.un.org/goals, accessed on 25 October 2021), particularly SDG 12 on responsible consumption and production. However, the health implications from the transition to circular economy are still to be explored in detail since it might stimulate the increase of risks of unintended adverse health effects, particularly for workers and related to managing risks from exposures to hazardous materials and waste [25]. Additionally, exposure assessment methods and data to assess the quantitative relationship between waste management and health effects are still limited [25].

Different matrices were used depending of the environment to be assessed. Passive sampling methods were the most commonly used, since they provide more time-integrated information and, because of that, they are more representative of the real exposure scenario [8,11,14,17,21]. Furthermore, the same extracts obtained from the matrices allow to combine different assays focusing on the targeted pollutants and the used cell lines [11,13,14,15,16,17,21].

Different cell lines were used depending on the environment assessed, exposure routes and target cells of some of the pollutants present in the mixture. A549 alveolar epithelial cancer-derived cells were used commonly for studies aiming to evaluate the toxicity of airborne mixtures. Although the A549 alveolar cell line has been widely used for over 40 years, inconsistencies remain as to its suitability as an appropriate model for type II primary alveolar cells, greatly depending on culture conditions for differentiation [26]. Indeed, the A549 cells have been used to model the alveolar type II pulmonary epithelium [27], for studying the metabolic processing of lung tissue and for identifying mechanisms of xenobiotics delivery to the tissue [28]. Type I alveolar epithelium is composed of thin cells, contributing about 95% of the alveolar surface in which the passive gas exchange takes place. Type II cells are large cuboidal cells that produce surfactant, occurring more diffusely (2-fold higher) than type I cells [28]. In addition, type II lung cells contain P450 isozymes suggesting a possible role in the oxidative metabolism of xenobiotics in the lung [29]. Another notable feature of type II cells is their endocytic properties [30,31], making them a potential target for delivery of macromolecules. According to our results, they were used both for active and passive air sampling.

Regarding the results for use of human bronchial epithelial cells (in three studies of occupational [4], indoor [18], and outdoor environments [19]), BEAS-2B cells are among the most used immortalized human bronchial/lung cell lines, being commonly used in studies of long-term exposure to metals [32]. A limitation of BEAS-2B is their inability to express MUC5AC, a secreted protein involved in mucociliary clearance that is constitutive of in vivo airway epithelium [33]. Indeed, immortalized or cancer-derived cells often present disrupted differentiation or lack crucial biomarkers typical of primary human adult cells due to their transformation. As for normal human bronchial epithelial (NHBE) cells, their use as a suitable in vitro model of bronchial epithelial cells has been recently reported [34]. These primary cells maintained normal epithelial phenotypic characteristics after four passages, including crucial CFTR ion channel function, which is important for airway mucociliary clearance. Primary cells can be further used to develop 3D primary human airway epithelial cultures as an in vitro model for toxicological assessments, minimizing animal experimentation, although 3D models are less available and expensive [34].

THP-1 (a human leukemia monocytic cell line) and human umbilical vein endothelial cells (HUVEC) cells (reported in two studies of waste sorting [8,17] and kitchen environments [20]) are well described in vitro cell models for immune modulation approach [35] and nanotoxicity assessments in the endothelium [36], respectively.

Finally, swine kidney cells, cited in three studies in the waste industry [11,14,17], and hepatocellular/Hep G2 cells, cited in one study in the same environment [17], were used to test the in vitro cytotoxicity of active and passive samples collected from the waste sorting industry. Renal cells are widely used for mycotoxin assessment due to their high sensitivity to mycotoxins [37,38]. As for hepatocellular/Hep G2 cells, cited in one study [17], they are non-tumorigenic cells with high proliferation rates and an epithelial-like morphology that perform many differentiated hepatic functions. They are commonly used in drug metabolism and hepatotoxicity studies [39,40,41]. Jennen and colleagues compared HepG2 with a novel human hepatoma cell line, HepaRG, for the purpose of chemical hazard identification, as an alternative to current rodent bioassays, and concluded that HepaRG was a more suited in vitro liver model for biological interpretations of the effects of exposure to chemicals, whereas HepG2 was a more promising in vitro liver model for classification studies using the toxicogenomics approach [42]. In contrast to HepG2, HepaRG cells can become highly differentiated (under specific culture conditions) and express various cytochrome P450 (CYP450) enzymes at a higher level and exhibit other hepatic metabolic functions, being more similar to cultured primary human hepatocytes [42]. For that reason, more recent studies conclude on HepaRG use as preferable for CYP induction studies [43,44], while not clear about their contribute to improve the early detection of drug-induced hepatotoxicity [43].

Some suggestions on how to include cytotoxicity assessment in risk characterization and management were already performed: a) using A549 as a model for the human alveolar epithelia, and swine kidney as renal cells) with the MTT assay to evaluate microbial contamination and to demonstrate whether filtering respiratory protection devices used in a variety of settings are adequate for human protection [11] and b) performing the overall cytotoxicity study from the samples extracts as a cytotoxicity pre-screening [11,14,21], following the identification of the potential indicators of each kind of pollutants—*Aspergillus* section *Fumigati* for mycological contamination in waste sorting industry for instance—and assess of their own contribution to the overall cytotoxicity [11].

From a more general perspective there is a need to consider the main challenges when using in vitro approaches which are the difficulties in simulating the consequences of long term exposures commonly occurring in workplaces [45]. However, the developments made in new assays allow generating enhanced information on dose-response relationships over a much wider range of concentrations, including those representatives of human exposure. The high-throughput methods now becoming more common will allow the expansion of the methods to larger numbers of endpoints, wider dose ranges, and mixtures of agents [46,47]. Research is ongoing to better understand how the dose-response relationships for perturbations might change with the duration of exposure and to understand pathway activation under acute, subchronic, and chronic exposure conditions [48]. However, single assays are not comprehensive or predictive in isolation but should be combined with other allowing more comprehensive understanding of the toxicity process. The development and assessment of batteries of assays would be desirable also for the context of exposures in workplaces such has been developed for other contexts (e.g., registration of a pesticide for food applications) [48].

Though, for the different risk contexts and decisions to be made the preferred test batteries may differ in sensitivity and specificity. Therefore, the validation of tests and test strategies for incorporation into assessment guidelines that will provide support on interpreting and making conclusions from the different test batteries results is of great importance [48].

## 5. Conclusions

Humans are commonly exposed to environmental contaminant mixtures that result in different toxicity than exposure to the single contaminants individually. This is the common exposure scenario in workplaces. Cytotoxicity assessment in environmental samples should have a more prominent role due to their contribution for identifying and better understanding the possible associations between co-exposure to environmental contaminants and adverse human health effects as a prioritization for the risk management. Indeed, cytotoxicity assessments may unveil the resulting interactions between the contaminants present and the toxicity resulting from this exposure. Future studies should focus on the validation of in vitro approaches for application in workplace exposure scenarios to allow the inclusion in assessment guidelines and make the results easier to interpret.

## Figures and Tables

**Table 1:** Inclusion and exclusion criteria.

Inclusion Criteria	Exclusion Criteria
Articles published in the English language	Articles published in other languages
Articles published from the 1 January 2000 to the 31 May 2021	Articles published prior to the 1 January 2000
Articles related to cytotoxicity in environmental samples or environmental isolates	Articles related exclusively to biological samples
Original scientific articles	Abstracts of congress, reports, reviews/state of the art articles

**Table 2:** Data selected from the chosen papers.

Database	Title	Country	Analyzed Environment	Samples Description/ Number of Samples	Cell Line Applied	Main Findings	References
**Web of Science**	The effects of waste sorting in environmental microbiome, THP-1 cell viability and inflammatory responses	Portugal	Occupational environment: waste sorting environment	Seventeen filters from the filtration system of forklifts operating in one waste sorting facility	Human monocytic THP-1 cells	Seven filters (39%) exhibited low or moderate cytotoxicity. The highest cytotoxic responses had a reduction in cell viability between 17 and 22%. Filter samples evoked proinflammatory responses (TNFα production).	[17]
**PubMed**	The London Underground: dust and hazards to health	UK	Outdoor environment: underground	PM2.5 samples collected using a portable DustTrak light scattering monitor in three busy London underground Stations; the particle number concentration (PNC) was measured using a P-Trak monitor.	Alveolar epithelial cell line A549	Cytotoxic and inflammatory potential at high doses, consistent with its composition largely of iron oxide (dust comprised by mass approximately 67% iron oxide, 1–2% quartz, and traces of other metals).	[13]
	Biomonitoring of Cyanobacterial Blooms in Polish Water Reservoir and the Cytotoxicity and Genotoxicity of Selected Cyanobacterial Extracts	Poland	Outdoor environment: water reservoir	Samples of blooms and water (1L) were collected during the intensive bloom and after decomposition of blooms.	Human lymphocytes	The cyanobacterial extracts at the beginning of September were most toxic to human lymphocytes (concentration of microcystins in water can increase to > 4 μg/L). The level of DNA damage in lymphocytes after short exposure to microcystic extracts (3 and 6 h) was significantly higher than respective levels after longer exposure.	[16]
**PubMed**	Use of Human Bronchial Epithelial Cells (BEAS-2B) to Study Immunological Markers Resulting from Exposure to PM_2_._5_ Organic Extract from Puerto Rico	USA	Outdoor environment: urban environment	PM_2_._5_ samples collected using a Fine Particulate Chemical Speciation Air Sampler at 17 L/min. Each filter represents the material collected in a 72 h sampling period at two different sites (Guaynabo and Fajardo).	Human bronchial epithelial BEAS-2B cells	Concentration of PM_2_._5_ collected at Guaynabo site was 10.982 μg/m^3^, 40.06% higher than in Fajardo—7.890 μg/m^3^. Organic PM_2_._5_ found to be a toxic and bioactive component that can regulate the secretion of cytokines in BEAS-2B.	[19]
	Differential Response of Human Nasal and Bronchial Epithelial Cells upon Exposure to Size-fractionated Dairy Dust	USA	Occupational environment: dairy farm environment	Airborne dust from a local dairy parlor was sampled and segregated by size using a high-volume cascade impactor over the course of single 72 h period at a flowrate of 1500 L/min. PM3 collected downstream of the impactor on an 8″ × 11″ Teflon filter replaced after 12 h.	Normal human bronchial epithelial (NHBE) and human nasal epithelial (HNE)	Both PM_10_ and PM_>10_ size fractions elicit a pro-inflammatory response in airway epithelial cells. NHBE respond differently to these dusts than HNE and, the two cell types need to be considered separately in airway cell models of agricultural dust toxicity.	[4]
	ROS-AKT-mTO R axis mediates autophagy of human umbilical vein endothelial cells induced by cooking oil fumes-derived fine particulate matters in vitro	China	Indoor environment: laboratorial simulation of a Chinese kitchen	COFs-derived PM_2_._5_ measurements in the laboratory: 200 mL peanut oil were poured and heated to smoke. Fumes were collected with filter paper connected to a total suspended particulates sampler filter that was renewed every 2 h.	Human umbilical vein endothelial cells (HUVEC)	When treated with 50, 100, and 200 μg/mL COFs-derived PM_2_._5_ for 12, 24, and 36 h, the cell viability were significantly lower than in the control group. COFs-derived PM_2_._5_ dose-dependent reduced the viability of HUVECs and increased the ROS levels in the cells.	[20]
	Environmental risk assessment of wastewaters from printed circuit board production: A multibiomarker approach using human cells	Croatia	Occupational environment/ Outdoor environment: wastewater contamination	Sixty L of wastewater was taken from a wastewater collecting tank from an advanced energy company. Blood sample from one donor.	Human peripheral blood lymphocytes	In the longer exposure period (24 h), survival significantly dropped by 33.22% in the untreated PCBW sample and by 25.52% in partially purified wastewater compared to the corresponding control sample, proving to be cytotoxic and genotoxic to human blood peripheral lymphocytes in vitro.	[15]
	Cytotoxic and Inflammatory Potential of Air Samples from Occupational Settings with Exposure to Organic Dust	Portugal	Occupational environment: poultry feed industry, swine feed industry, waste sorting plant, poultry pavilion and slaughterhouse	Air samples collected by the impinger method (300 L samples collected at 300 L/min airflow rate. PM_2_._5_ samples were collected for 30-min from each location (2 L/min flow rate).	Human monocytic THP-1 cells	Air samples collected from the assessed workplaces caused both cytotoxic and pro-inflammatory effects. Viability of the cells in the swine feed industry was only 20%.	[8]
**PubMed**	The pro-inflammatory effects of particulate matter on epithelial cells are associated with elemental composition	Australia	Indoor environment: house environment	A minimum weight of 20 mg was collected using a HVS4 US EPA approved vacuum sampler from 36 homes of non-smokers in suburban Victoria	Human bronchial epithelial BEAS-2B cells	Using an approximate conversion of 10 EU/ng, cells were exposed to an average of 0.05 ng endotoxin in the high dose group. Positive associations between pro-inflammatory effects of roof space PM samples with Fe, Al, and Mn levels (84.43%).	[18]
	Cytotoxicity Assessment of PM_2_._5_ Collected from Specific Anthropogenic Activities in Taiwan	Taiwan	Outdoor environment: traffic	PM_2_._5_ samples collected at long-range transport. Traffic stations were obtained from 24-h sampling and night market samples were only collected for 6 h at a flow rate of 500 Lmin^−1^. The high-volume samplers for PM_2_._5_ captured particles on quartz fiber filters (two filters per station).	Alveolar epithelial cell line A549	Cell viability reduced to 9% after exposure to organic extracts of 0.316 μg of PM_2_._5_ from LRT and night market samples. Organic extracts from night market induced positive genotoxicity in umu test (at a dose of 20.0 μg PM_2_._5_).	[12]
	Cytotoxic effect of Filtering respiratory protective devices from the waste sorting industry: is in vitro toxicology useful for risk characterization?	Portugal	Occupational environment: waste sorting industry	118 FRPD sampled (feeding machines with waste (*n* = 33), sorting waste (*n* = 54), machine inspection (*n* = 12), machines and special vehicles operator (*n* = 13), and FRPD from non-identified workstations (*n* = 8))	Alveolar epithelial cell line A549 and swine kidney cells	Cytotoxic effect in A549 cells, of which 81 presented high cytotoxicity. In SK cells, a cytotoxic effect was observed in 56 samples, of which five displayed a high cytotoxic effect.	[14]
	Assessment of the microbial contamination of mechanical protection gloves used on waste sorting industry: A contribution for the risk characterization	Portugal	Occupational environment: waste sorting industry	Sixty seven mechanical protection gloves (MPG) sampled (feeding machines with waste (*n* = 9), sorting waste (*n* = 40), machine inspection (*n* = 10), and machines and special vehicles operator (*n* = 8))	Swine kidney cells and hepatocellular carcinoma (Hep G2)	The most reported mycotoxin was mycophenolic acid (89.6%). HepG2 cells appear to be more sensitive to MPG contamination, with high cytotoxicity (IC50 < 0.05 mm^2^/mL) observed for 18/57 gloves.	[21]
	Cytotoxicity of filtering respiratory protective devices from the waste sorting industry: A comparative study between interior layer and exhalation valve	Portugal	Occupational environment: waste sorting industry	118 FRPD sampled (feeding machines with waste (*n* = 33), sorting waste (*n* = 54), machine inspection (*n* = 12), machines and special vehicles operator (*n* = 13), and FRPD from non-identified workstations (*n* = 8))	Alveolar epithelial cell line A549 and swine kidney cells	50% inhibitory concentration (IC_50_) values lower for FRPD interior layer than exhalation valves in lung cells. Higher bacterial counts in TSA were correlated with lower IC_50_ values, thus, higher cytotoxicity effect in lung cells.	[11]

## Supplementary Materials

The following are available online at https://www.mdpi.com/article/10.3390/toxics10020072/s1, Table S1: PRISMA Checklist (Adopted from [1]).
