# Supplementary material for: Are In Vitro Cytotoxicity Assessments of Environmental Samples Useful for Characterizing the Risk of Exposure to Multiple Contaminants at the Workplace? A Systematic Review
Source: Toxics. 2022 Feb 5;10(2):72. doi: 10.3390/toxics10020072 (PMC8879481; doi:10.3390/toxics10020072)
Supplement: Supplementary file 1 [file toxics-10-00072-s001.zip › toxics-1563030-supplementary.pdf]

toxics MDPI

Supplementary Materials: Are In Vitro Cytotoxicity Assessments of Environmental Samples Useful for Characterizing the Risk of Exposure to Multiple Contaminants at the Workplace? A Systematic Review

Carla Viegas, Pedro Pena, Bianca Gomes, Marta Dias, Liliana Aranha Caetano, Susana Viegas

Table S1. Table S1 – PRISMA Checklist (Adopted from [1])

Section/Topic	#	Checklist Item	Reported on Page #
**TITLE**			
Title	1	Identify the report as a systematic review, meta-analyses	1
**ABSTRACT**			
Structured summary	2	Provide a structured summary including, as applicable: background; objectives; data sources;study eligibility criteria, participants, and interventions; study appraisal and synthesis methods; results; limitations; conclusions and implications of key findings; systematic review registration number.	1
**INTRODUCTION**			
Rationale	3	Describe the rationale for the review in the context of what is already known.	1 and 2
Objectives	4	Provide an explicit statement of questions being addressed with reference to participants, interventions, comparisons, outcomes, and studydesign (PICOS).	2
**METHODS**			
Protocol and registration	5	Indicate if a review protocol exists, if and where it can be accessed (e.g., Web address), and, if available, provide registration information includingregistration number.	2
Eligibility criteria	6	Specify study characteristics (e.g., PICOS, length offollow-up) and report characteristics (e.g., years considered, language, publication status) used as criteria for eligibility, giving rationale.	2
Information sources	7	Describe all information sources (e.g., databases with dates of coverage, contact with study authors to identify additional studies) in the search and datelast searched.	2
Search	8	Present full electronic search strategy for at least one database, including any limits used, such that itcould be repeated.	3

Study selection	9	State the process for selecting studies (i.e., screening, eligibility, included in systematic review,and, if applicable, included in the meta-analysis).	3
Data collection process	10	Describe method of data extraction from reports (e.g., piloted forms, independently, in duplicate) andany processes for obtaining and confirming data from investigators.	3
Data items	11	List and define all variables for which data weresought (e.g., PICOS, funding sources) and any assumptions and simplifications made.	3
Risk of bias inindividual studies	12	Describe methods used for assessing risk of bias of individual studies (including specification of whetherthis was done at the study or outcome level), and how this information is to be used in any data synthesis.	3
Summary measures	13	State the principal summary measures (e.g., riskratio, difference in means).	3
Synthesis of results	14	Describe the methods of handling data and combining results of studies, if done, including measures of consistency (e.g., I^2) for each meta-analysis.	3
Risk of bias across studies	15	Specify any assessment of risk of bias that may affect the cumulative evidence (e.g., publicationbias, selective reporting within studies).	3
Additional analyses	16	Describe methods of additional analyses (e.g., sensitivity or subgroup analyses, meta-regression), ifdone, indicating which were pre-specified.	3
**RESULTS**			
Study selection	17	Give numbers of studies screened, assessed for eligibility, and included in the review, with reasonsfor exclusions at each stage, ideally with a flow diagram.	3, Figure 1
Study characteristics	18	For each study, present characteristics for which data were extracted (e.g., study size, PICOS, follow-up period) and provide the citations.	4 and 5, Table 2
Risk of bias within studies	19	Present data on risk of bias of each study and, if available, any outcome level assessment (see item12).	3
Results of individual studies	20	For all outcomes considered (benefits or harms), present, for each study: (a) simple summary data foreach intervention group (b) effect estimates and confidence intervals, ideally with a forest plot.	Table 2
Synthesis of results	21	Present results of each meta-analysis done, including confidence intervals and measures ofconsistency.	Table 2

Risk of bias across studies	22	Present results of any assessment of risk of biasacross studies (see Item 15).	3
Additional analysis	23	Give results of additional analyses, if done (e.g., sensitivity or subgroup analyses, meta-regression[see Item 16]).	Not applicable
**DISCUSSION**			
Summary of evidence	24	Summarize the main findings including the strengthof evidence for each main outcome; consider their relevance to key groups (e.g., healthcare providers, users, and policy makers).	9 and 10
Limitations	25	Discuss limitations at study and outcome level (e.g.,risk of bias), and at review-level (e.g., incomplete retrieval of identified research, reporting bias).	10
Conclusions	26	Provide a general interpretation of the results in thecontext of other evidence, and implications for future research.	9 and 10
**FUNDING**			
Funding	27	Describe sources of funding for the systematic review and other support (e.g., supply of data); roleof funders for the systematic review.	11

References

1. Moher, D.; Liberati, A.; Tetzlaff, J.; Altman, D.G., The PRISMA Group (2009). Preferred Reporting Items for Systematic Reviews and Meta-Analyses: The PRISMA Statement. PLoS Med, **2009**, *6*(7): e1000097. doi:10.1371/journal.pmed1000097.
